# Diet in Disease

**Published:** 1893-06-17

**Authors:** Ernest Hart

**Affiliations:** Bachelier-ès-Sciences-es-Lettres (rèstreint), formerly Student of the Faculty of Medicine of Paris, and of the London School of Medicine for Women


### DIET IN DISEASE.

BY MRS. ERNEST HART, Bachelier-ès-Sciences-es-Lettres (rèstreint), formerly Student of the Faculty of Medicine of Paris, and of the London School of Medicine for Women.

### XXIII.—FOOD FOR THE AGED.

THOUGH old age may not be considered in the light of a disease, yet, as there is at this period a general enfeeblement of the powers, I do not think it will be out of place if after having insisted, as I did in the last paper, upon the necessity of diminishing the intake of food, particularly of albuminous foods, in old age, I give a few recipes to show how the aged may be well fed on a light diet. Fish and poultry should take the place of butcher's meat. Besides the ordinary methods of boiling, frying and baking fish, soups and delicate dishes may be made of fish, which will be found to be not only appetising but satisfying to those on whose muscular powers but slight demands are made. Fish contains a third less of albuminoids than ordinary meat, and is hence very suitable as an article of diet for the old. Sir Henry Thompson, to whose scientific and practical studies on food we owe so much, points out that, besides the well-known sole, turbot, salmon, whiting, haddock, mackerel, cod, trout, smelt, herring, skate, and mullet, there are other kinds of fish admirable for food, which yet are almost totally neglected by the British housewife. These are the wolf-fish or cat-fish, the halibut, sea bream, bass, gurnet, ling, hake, thornback, pollock, and coal-fish, to which may be added the conger, excellent for making soups and stews, and the sturgeon (of which the flesh approaches that of meat in quality). The following are Sir Henry Thompson's recipes for fish soup :—

1. Put three ounces of butter into a stewpan, add two carrots sliced, one onion, and a shalot in thin slices, then cloves, a little thyme, and some parsley. Fry them gently until of a reddish tint, then add three pints of cold water. Let it boil, skimming occasionally. Then add a small fresh haddock, bones and all, cut up into pieces, and the head and bones of two whitings, setting aside the fillets ; a cod's head or that of a turbot, or the fresh bones, head, and fins of two large soles, the fillets of which are required for another dish, may take the place of the foregoing. Add some salt and a little pepper. Let all simmer together for two hours gently at the corner of the fire ; take out the bones, and pass all the rest through a coarse strainer. Divide the fillets of whiting into two or three small portions each ; boil for a few minutes in some of the stock, add a little fresh green chervil and parsley chopped not too finely, and serve all together in a tureen. This soup may be thickened if desired by adding a tablespoonful of white "roux," that is a little flour well mixed with butter in a stewpan over the fire, cooked but not allowed to brown. This is unquestionably an improvement. Fillets of other fish may be substituted for those of the whiting, or a few shell fish or oysters if they are well digested.

2. The following is the receipt for an economical fish stew :—

Take three or four pounds of hake, ling, skate, or haddock, and one pound of "cuttings or trimmings," which are the best part of the fish for stock making. Remove all the fish from the bones, break up or pound the latter, and set aside with any portion of head there may be and the cuttings. Put into a saucepan over the fire two ounces of lard and two or three onions sliced, and let them fry until brown ; then add two quarts of water and all the pounded bones and trimmings, some parsley or other green herbs, pepper and salt. Let the whole simmer for three hours, adding the amount of water lost by evaporation. Strain out the bones, bits of skin, &c., add the fish in pieces, and boil gently ten or fifteen minutes. Thicken with sufficient flour mixed smoothly with a small portion of stock, and added before finishing. In order to make the dish complete and substantial a few small suet dumplings should be well boiled and put into the tureen.

### STEWED COD.

Have ready some boiling water in a saucepan and put a little salt in it. Take a slice of cod about an inch thick and half a pound in weight. Clean it and put it into the boiling water and let it boil gently for five minutes ; then lift it out and let it drain. Have ready heated in a stewpan one gill of veal gravy or good broth. Put the cod in this and stew it for five minutes ; then add a tablespoonful of very fine bread crumbs, and let it simmer for three minutes. Mix a teaspoonful of arrowroot and half a teaspoonful of anchovy sauce, with a dessertspoonful of sherry, and a teaspoonful of lemon juice, and stir it well into the gravy. Boil all together for two minutes, then lift the fish out carefully with a fish slice. Pour the sauce over it and serve it quickly. Half a dozen oysters, bearded and added with their strained liquor two or three minutes before the cod is taken out of the stew-pan improve this dish.

### HADDOCK PUDDING.

Boil a haddock weighing about one pound for about ten to fifteen minutes in boiling water with a little salt and a tablespoonful of vinegar. Remove all the skin and bones and cut the fish in small pieces. Boil half a pound of potatoes in salt and water until they are soft, then rub them through a sieve and mix them with the fish. Add one raw egg, an ounce of butter, and a little pepper and salt. When thoroughly mixed make the compound into any shape preferred ; put it into a buttered tin and bake it until it is of a golden colour. Serve the pudding with egg sauce made as follows : Mix one ounce of butter with one ounce of flour in a saucepan over the fire. Add gradually one gill of the water in which the haddock was boiled, and one gill of milk. Stir over the fire for ten minutes ; then add two hard-boiled eggs which have been cut into very small dice, and a few drops of lemon juice. Pour this sauce round the fish pudding.

### MACARONI AND FISH.

Cut a quarter of a pound of well-boiled macaroni into small pieces. Take away the skin and bones of a quarter of pound of cold boiled fish. Mix the macaroni and fish well together, with a little pepper and salt, half a pint of good fish or chicken broth, and one ounce of butter. Put the mixture into the oven, and when it is quite hot and brown it will be ready to serve.

### KEDGEREE.

Warm in a saucepan, stirring all the time, a quarter of a pound of cooked fish, a quarter of a pound of rice after it has been boiled, and one ounce of butter. Beat up one egg, with a little pepper and salt. Add it to the fish and rice, and cook all together for two minutes. If it be too stiff, add a little milk.

### THE POT-AU-FEU.

The pot-au-feu as prepared in France is savoury and nutritious. The following recipe is adapted from "Le Livre de la Cuisine," by T. Gouffe :—

In a tin-lined iron or copper pot, place about two pounds of the leg or shoulder of beef, and half a pound of bones broken into fragments, in four quarts of cold water. The bones should be put in first, then the meat tied up to preserve its shape, add one ounce of salt, place the pot over a steady clear fire which will give a constant gentle heat, bring the water to the boil and skim carefully. As soon as the scum rises pour in a little cold water ; let the water boil three separate times, skimming each time. Then add the vegetables, which should consist of a pound of cut carrots, onions, and turnips, half a pound of leeks, an ounce of parsnips, half an ounce of celery, and three cloves stuck in an onion. The throwing in of the vegetables will temporarily check the boiling. As soon as the water is brought to the boil again, draw the pot aside and place it on a spot on the fire or the hot plate where it will simmer gently and steadily for three hours. The vegetables should be left only just long enough in the broth to cook them. When done the meat is withdrawn, and while still on the fire the broth is freed perfectly from grease. In France this thoroughly-boiled beef is eaten as a separate dish, either hot with the vegetables, or cold served with oil and vinegar. The broth is frequently served with *croutons* of toast, or with the leaves of boiled spring cabbage floating in the tureen.

Sweetbread Soup.

Boil a pair of sweet-breads for five minutes with a little water; skin, trim, and boil them gently in one and a half pints of white stock, with a bouquet of herbs, a piece of celery, or as much celery seed as will lie on a three-penny piece, and a shred of mace until they are quite tender. When they are quite soft either pass them through a hair sieve or chop them finely. Remove the herbs, add a little pepper and salt, a few drops of lemon juice, and a gill of cream.

Brunoise Soup.

Take one young carrot, half a young turnip, two leaves of celery, a little of the flower of a boiled cauliflower, one onion, one ounce of butter, one pint of water in which the cauliflower was boiled, one pint of milk, one teaspoonful of salt, pepper and salt, and two ounces of stale bread toasted. Stew the ingredients, except the toast, together for one hour; then break the toast in pieces, add it to the rest and stew all together for another hour. Pass all through a sieve and return it to the stew-pan to get hot.

Maigre Soup.

Shred one pound of potatoes and put them with one leek, one onion, and one ounce of butter into a pint of boiling water in a stew-pan. Boil until the vegetables are soft, then pass them through a sieve, adding a pint of hot milk to help them through Put all into the stew-pan, and stir until it boils, then sprinkle in one tablespoonful of Groult's tapioca. Boil until the tapioca is clear; flavour with a little ground mace, pepper and salt, add a little lemon juice, and a table-spoonful of chopped parsley.

Steamed Asparagus.

Trim the asparagus, then steam it by putting it in a jam-pot nearly filled with boiling water, placed in a large saucepan half full of boiling water and tightly covered. It will take nearly an hour to cook in this manner. Serve with the asparagus a sauce made of one ounce of butter melted over the fire, one tablespoonful of cream, the yolk of an egg, and five drops of lemon juice. Stir the mixture in an enamelled saucepan over the fire for three minutes.

Apple Snowballs.

Boil half a pound of the best rice in boiling water for fifteen or twenty minutes. Strain it and spread it on floured cloths. Peel and core one or two apples and put them on the rice. Sprinkle over them sugar and a little lemon juice, then cover each one entirely with rice, tie the cloths, and boil them for one hour.

For many of these practicable recipes I am indebted to that excellent and valuable manual on "The Art of Feeding the Invalid." They might be multiplied indefinitely. The principle to be remembered is that the food of the aged should be light, farinaceous, and easily digested. Among things to be recommended, I might mention besides all kinds of fish, rice, tapioca, arrowroot, sago, custard, and bread and butter puddings, poultry, game, fresh vegetables, ripe fruit, omelette, junket, and milk. The food of extreme old age compares with that of extreme youth, and for toothless age pap is as useful as to the teething babe, nor must it be thought that the dentist's art gives the stomach the power to digest the strong meats suitable to youth.

